# Motor Synchronization to Rhythmic Auditory Stimulation (RAS) Attenuates Dopaminergic Responses in Ventral Striatum in Young Healthy Adults: [^11^C]-(+)-PHNO PET Study

**DOI:** 10.3389/fnins.2019.00106

**Published:** 2019-02-14

**Authors:** Yuko Koshimori, Antonio P. Strafella, Mikaeel Valli, Vivek Sharma, Sang-soo Cho, Sylvain Houle, Michael H. Thaut

**Affiliations:** ^1^Music and Health Research Collaboratory, Faculty of Music, University of Toronto, Toronto, ON, Canada; ^2^Research Imaging Centre, Campbell Family Mental Health Research Institute, Centre for Addiction and Mental Health, University of Toronto, Toronto, ON, Canada; ^3^Division of Brain, Imaging and Behaviour – Systems Neuroscience, Krembil Research Institute, University Health Network, University of Toronto, Toronto, ON, Canada; ^4^Morton and Gloria Shulman Movement Disorders Clinic and The Edmond J. Safra Program in Parkinson’s Disease, Neurology Division, Department of Medicine, Toronto Western Hospital, University Health Network, University of Toronto, Toronto, ON, Canada; ^5^Baycrest Health Sciences, Rotman Research Institute, Toronto, ON, Canada

**Keywords:** finger tapping, rhythmic auditory stimulation, D2/3 receptors, dopamine, PET, [^11^C]-(+)-PHNO, auditory-motor entrainment, basal ganglia

## Abstract

Auditory-motor entrainment using rhythmic auditory stimulation (RAS) has been shown to improve motor control in healthy persons and persons with neurologic motor disorders such as Parkinson’s disease and stroke. Neuroimaging studies have shown the modulation of corticostriatal activity in response to RAS. However, the underlying neurochemical mechanisms for auditory-motor entrainment are unknown. The current study aimed to investigate RAS-induced dopamine (DA) responses in basal ganglia (BG) during finger tapping tasks combined with [^11^C]-(+)-PHNO-PET in eight right-handed young healthy participants. Each participant underwent two PET scans with and without RAS. Binding potential relative to the non-displaceable compartment (BP_ND_) values were derived using the simplified reference tissue method. The task performance was measured using absolute tapping period error and its standard deviation. We found that the presence of RAS significantly improved the task performance compared to the absence of RAS, demonstrated by reductions in the absolute tapping period error (*p* = 0.007) and its variability (*p* = 0.006). We also found that (1) the presence of RAS reduced the BG BP_ND_ variability (*p* = 0.013) and (2) the absence of RAS resulted in a greater DA response in the left ventral striatum (VS) compared to the presence of RAS (*p* = 0.003), These suggest that the absence of external cueing may require more DA response in the left VS associated with more motivational and sustained attentional efforts to perform the task. Additionally, we demonstrated significant age effects on D2/3 R availability in BG: increasing age was associated with reduced D2/3 R availability in the left putamen without RAS (*p* = 0.026) as well as in the right VS with RAS (*p* = 0.02). This is the first study to demonstrate the relationships among RAS, DA response/D2/3 R availability, motor responses and age, providing the groundwork for future studies to explore mechanisms for auditory-motor entrainment in healthy elderly and patients with dopamine-based movement disorders.

## Introduction

Rhythmic auditory stimulation (RAS) – presented either as single auditory beats or metronome clicks embedded in instrumental music – has shown to improve motor control in healthy persons and persons with neurologic motor disorders such as Parkinson’s disease (PD) and stroke (Miller et al., 1996; Thaut et al., 1996, 2002; McIntosh et al., 1997; Massie et al., 2009). Reduction in variability of motor timing, electromyography recruitment, and movement kinematics as well as increases in speed are among the positive effects demonstrated.

These benefits result from rhythmic auditory entrainment. Entrainment refers to the frequency locking of two oscillating bodies that can move in stable periodic cycles (Thaut, 2015). The rhythmic frequency provides the brain (already equipped with internal time keeper mechanism) with an additional externally triggered time keeper, which generates a precise temporal interval as a continuous time reference (Thaut, 2015). Importantly, the auditory system is more precise and faster to detect temporal patterns than other sensory systems such as visual and tactile systems (Shelton and Kumar, 2010).

Auditory rhythm can prime and time muscle activation by providing precise anticipatory time cues for motor planning and execution (Paltsev and E-lner, 1967; Rossignol and Jones, 1976), which increases the readiness to move and improves subsequent response quality (Thaut, 2015). Once auditory-motor entrainment occurs, movements stays locked to the auditory rhythm presented even when subtle tempo changes occur in the auditory stimuli that are not consciously perceived (Thaut et al., 1998a,b; Large et al., 2002).

The auditory and the motor systems are connected through widely distributed and hierarchically organized neural networks from cortical to subcortical, brain stem, and cerebellar regions (Thaut, 2003; Schmahmann and Pandya, 2006; Felix et al., 2011; Konoike et al., 2012). Functional MRI studies have shown that listening to regular auditory rhythm modulated activities in premotor (Chen et al., 2006, 2008), cortico-basal ganglia (BG) including putamen, caudate, and pallidum (Grahn and Brett, 2007; Grahn and Rowe, 2009, 2013), and cortico-cerebellar (Thaut et al., 2009; Konoike et al., 2012) networks. It also led to the rapid and precise brain wave entrainment, mainly in beta oscillation bands in the motor areas such as supplementary motor area (SMA) and cerebellum (Fujioka et al., 2012; Crasta et al., 2018). In addition to auditory rhythm, music generally modulates activity in widely distributed brain areas, particularly in the limbic regions including the nucleus accumbens/ventral striatum (VS) (Blood and Zatorre, 2001; Menon and Levitin, 2005; Salimpoor et al., 2011, 2013; Koelsch, 2014; Mueller et al., 2015). Furthermore, anatomical (Hackett, 2015) and resting functional MRI (Helmich et al., 2010) studies demonstrated connectivity between superior temporal gyrus and striatum (i.e., putamen and caudate). These suggest a close link between auditory areas and BG.

However, it is not well understood how dopamine (DA) in BG is involved in auditory-motor entrainment due to a paucity of research. Neuroimaging studies can investigate the neural mechanisms by employing a synchronization paradigm (Thaut et al., 1998a,b), in which finger tapping is synchronized to external auditory cueing that is thought to occur through entrainment (Braunlich et al., 2018). This can be contrasted to a continuation task (i.e., finger tapping without external auditory cueing) to elucidate the role of external auditory cueing in motor timing (Koshimori and Thaut, 2018; Teghil et al., 2018). To our knowledge, all of the studies using the synchronization/continuation task design employed fMRI and showed that cortical motor areas and cerebellum were activated during auditory-motor entrainment (Rao et al., 1997; Jäncke et al., 2000; Toyomura et al., 2012), but whose activation was similar to that during the continuation task. However, activation of the putamen and SMA was absent during the synchronization task (Rao et al., 1997; Lewis et al., 2004; Toyomura et al., 2012). These suggest that external auditory cueing may not have an extra role in brain activation in motor areas or that external auditory cueing may not require cortico-basal ganglia activity in young healthy adults. On the other hand, one PET study in PD suggested the association between dopaminergic function measured by baseline striatal dopaminergic denervation and auditory-motor synchronization performance (Miller et al., 2013). In addition, pharmacological studies in PD (McIntosh et al., 1997; Rochester et al., 2010) suggested that DA may play a partial role in auditory-motor entrainment or that intact dopaminergic system may be required for auditory-motor entrainment (Koshimori and Thaut, 2018).

To date there have been no studies investigating the role of BG DA in auditory-motor entrainment using the synchronization/continuation paradigm. Studying dopaminergic responses may be of particular importance to understand how PD benefits from RAS, since BG is an important subcortical structure for timing perception, which is crucial for movements and is negatively affected by dopamine depletion, yet persons with PD have significantly improved motor control with RAS, especially in gait performance (Ghai et al., 2018). The current study is therefore intended to investigate neurochemical mechanisms underlying the effect of RAS through dopamine responses in young healthy adults, serving as a baseline response to which later studies with healthy elderly and patients with dopamine-deficient movement disorders can be compared.

For this purpose, we employed [^11^C]-(+)-PHNO-PET to measure dopaminergic function during auditory-motor entrainment. Compared to the commonly used D2/3 R radioligand, [^11^C]-racropride, it shows higher sensitivity in detecting D3 receptors (Narendran et al., 2006), which allows for better quantification of the regions with greater expression of D3 receptors such as the ventral striatum (VS) and globus pallidus (GP) (Narendran et al., 2006; Willeit et al., 2006). In addition, being an agonist radioligand, it is more sensitive to competition with endogenous DA *in vivo* compared to an antagonist radioligand, [^11^C]-racropride (Caravaggio et al., 2014, 2016), which is more advantageous in a functional study. It has been employed in the studies that investigated task-induced functional changes (Mizrahi et al., 2014) and task-induced functional changes associated with reward and motivation (Caravaggio et al., 2018).

We hypothesized that (1) finger tapping performance would be better with RAS compared to No-RAS, (2) the PET outcome measure, BP_ND_ values would be significantly different between the task conditions in BG and its regions, and (3) finger tapping performance would be associated with BP_ND_ values.

## Materials and Methods

### Participants

Twelve right-handed healthy participants (seven women and five men) aged from 18 to 35 years enrolled in this study. The participants were recruited through self-referral in response to advertisements. Inclusion criteria were: no history of neurologic/psychiatric disorders or major medical conditions, no hearing issues, no contradictions for PET and MRI scans (e.g., metal implants, claustrophobia, pacemaker, or pregnancy), no alcohol, medication or drug dependency or abuse, right-handedness confirmed by the Edinburgh Handedness Inventory (Oldfield, 1971), no current depression assessed by Beck Depression Inventory II (BDI II). The study was carried out in accordance with the recommendations of the Ethical Committee of the Centre for Addiction and Mental Health. All participants gave written informed consent in accordance with the Declaration of Helsinki. The protocol was approved by the Ethical Committee of the Centre for Addiction and Mental Health.

### Study Design

**FIGURE 1:**
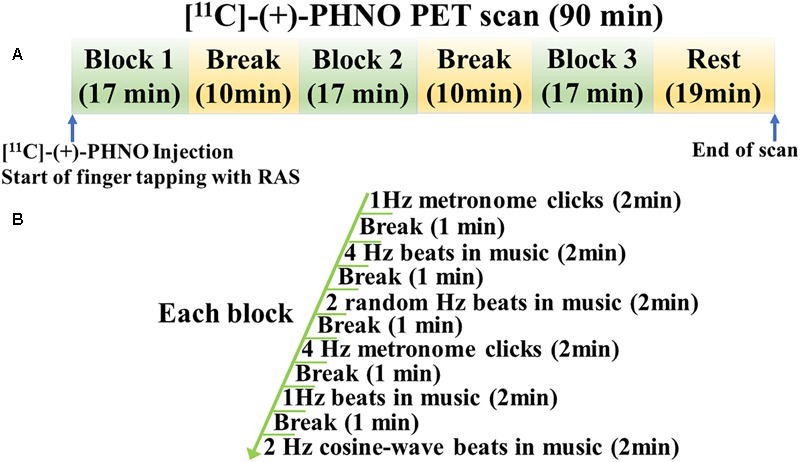
Study design for finger tapping task during PET scan. **(A)** PET scan consisted of three task blocks with two breaks in between followed by resting. **(B)** Each task block consisted of six tapping sessions with breaks in between, starting with 1 Hz metronome clicks. RAS was presented in the entire task block in the RAS condition whereas it was faded after six beats/clicks in the No-RAS condition.

Each participant underwent two [^11^C]-(+)-PHNO PET and one MRI scans. During one scan, a participant was performing a finger-tapping task with RAS (RAS condition) and during the other scan, a finger-tapping task without RAS (No-RAS condition). A finger tapping task started upon the radioligand injection to be consistent across the participants (Figure 1). During each scan, there were three task blocks with 10-min rests in between. One block consists of six tapping sessions with each lasting for 2 min followed by a 1-min rest. The total time of one task block lasted for 17 min. After completion of three task blocks, a participant rested until the PET scan was completed. The order of the two PET scans was counterbalanced across participants.

### Finger Tapping Task

Rhythmic auditory stimulation was programmed using Psychophysics Toolbox Version 3 (Brainard, 1997; Pelli, 1997; Kleiner et al., 2007) and run on Matlab (R2016a). It was presented through speakers above the participant’s head located outside of the scanner. The loudness of RAS was tested after the participant laid down on the scanner bed. It was set at a comfortable listening level determined by each participant. In addition, a keyboard on which the participant tapped was stabilized at a comfortable location. Participants performed the task briefly before the radioligand injection. Beep sounds indicated the start and the end of each tapping session. In the RAS condition, a participant was asked to tap his/her right index finger to six different rhythmic cues including (1) 1 Hz beats embedded in music (Capricho Catalán by Isaac Albéniz), (2) 4 Hz metronome clicks, (3) 2 Hz beats with random tempo changes below the threshold of conscious perception (4% of interbeat interval) embedded in music (Allegro Robusto by Bela Bartok), (4) 4 Hz beats embedded in music (Rondo a Capriccio “Rage Over My Lost Penny” by Ludwig von Beethoven), (5) 1 Hz metronome clicks, and (6) 2 Hz with tempo changes continuously modulated on a cosine function below the threshold of conscious perception (4% of interbeat interval) embedded in music (Allegro Robusto by Bela Bartok). Our choice of the auditory stimuli regarding the frequency and presentation mode was based on extensive research in synchronization paradigms using auditory rhythm and finger tapping in the past 50 years in healthy persons and persons with movement disorders: the most pronounced entrainment effects have been demonstrated ranging between 1 and 4 Hz (e.g., Michon and van der Valk, 1967; Rao et al., 1997; Thaut et al., 1998a,b; Stephan et al., 2002; Thaut and Kenyon, 2003; Molinari et al., 2005; Repp, 2005; Thaut et al., 2008; Braunlich et al., 2018). The current study aimed to investigate DA responses across the typical frequency range of entrainment. In order to enhance beat perception, a musical context via melody and harmony components was added to half of the rhythm presentation (Thaut et al., 1997; Nozaradan et al., 2011). The choice of the order and mode of presentation of RAS was also intended to reduce fatigue of the participants and boredom of the task. The RAS was presented in the same order across the participants. In the No-RAS condition, a participant was asked to tap his/her right index finger to the six cue-in beats/clicks following the same rhythm presentation and order as in the RAS condition. After six beats/clicks, the cue was faded, and participants continued to tap without the external cuing. Participants practiced each finger tapping task outside of the scanner on the scan day. The finger tapping performance was recorded on Matlab (R2016a) and measured using the absolute tapping period error and its standard deviation. The absolute tapping period error was the absolute differences between interstimulus intervals and interresponse intervals. The absolute tapping period errors that were 50% longer or shorter than the target intervals were excluded as outliers from the analysis. In addition, the first six taps were excluded from the analysis.

### MRI Acquisition

Each participant underwent one structural MRI scan acquired with a General Electric Discovery MR 750 3T scanner with 8-channel head coil (General Electric, Milwaukee, WI, United States). Proton density-weighted MRIs (oblique plane, 84 slices; matrix of 256 × 192; 22 cm field of view; 2 cm slice thickness; echo time = Min Full; repetition time = 6000 ms; flip angle = 8°) were used for co-registration to PET images for region of interest (ROI) delineation.

### PET Acquisition

Each participant underwent two [^11^C]-(+)-PHNO scans acquired with a high resolution PET/CT Siemens-Biograph HiRez XVI (Siemens Molecular Imaging, Knoxville, TN, United States), operating in 3D mode with an in-plane resolution of approximately 4.6 mm full width at half-maximum. The radiosynthesis of [^11^C]-(+)-PHNO ([^11^C]-(+)-4-propyl-9-hydroxynaphthoxazine) has been described in detail elsewhere (Wilson et al., 2005). Briefly, [^11^C]-propionyl chloride was reacted with 9-hydroxynaphthoxazine to generate a [^11^C]-amide, which is subsequently reduced by lithium aluminum hydride. Purification by HPLC and formulation give radiochemically pure [^11^C]-(+)-PHNO as a sterile, pyrogen-free solution suitable for human studies.

Prior to the PET scan, a low dose (0.2 mSv) CT scan was performed and used for attenuation correction. In order to prevent head movement during the PET scan, a thermoplastic facemask was custom-fitted to each participant and attached to a head-fixation system (Tru-Scan Imaging, Annapolis). For each scan, ∼10 mCi [^11^C]-(+)-PHNO was injected as a bolus into an intravenous line placed in an antecubital vein. The emission data were acquired over 90 min. For each 3D sinogram, data were normalized with attenuation and scatter corrected before applying fourier rebinning to convert the 3D sinograms into 2D sinograms. The 2D sinograms were then reconstructed into image space using a 2D filtered back projection algorithm, with a ramp filter at Nyquist cutoff frequency. After reconstruction, a Gaussian filter with a 5 mm FWHM was applied and the images calibrated to nCi/cc. The spatial resolution of the reconstructed images was 2 mm × 2 mm × 2 mm.

### PET Image Processing

[^11^C]-(+)-PHNO images were processed using in-house software ROMI platform (Rusjan et al., 2006). The preprocessing steps included (1) motion correction if necessary, (2) transformation of a standard brain template with a set of predefined ROIs to match individual high-resolution MR images, (3) refinement of the ROIs from the transformed template based on the gray matter probability of voxels in the individual MR images (segmentation step), (4) coregistration of the individual MR images to the PET images to transform the individual refined ROIs to the PET image space, and (5) extraction of time activity curves of the ROIs. We chose ROI analysis over voxel-based analysis due to poor spatial resolution of PET imaging. Our ROIs included the entire BG and its regions (i.e., putamen, caudate, VS, and GP). Binding potential relative to the non-displaceable compartment (BP_ND_) were extracted bilaterally in the ROIs using the simplified reference tissue model with the cerebellum (excluding the vermis) as a reference region using PMOD (version 3.6).

### Statistical Analysis

The normality was tested on the behavioral and [^11^C]-(+)-PHNO PET outcome measures using a Shapiro–Wilk test. Correlation analyses were performed between age and music experience, and BP_ND_ values. Depending on the results of these tests, appropriate statistical analyses were used to test differences in BP_ND_ values between conditions as well as correlations (1) between BP_ND_ values and task performance for each condition and (2) between percentage changes in BP_ND_ value and percentage changes in task performance. The statistical analyses were conducted using SPSS (version 20). The significance level for the statistical analyses was set at *p* < 0.05 (Bonferroni corrected).

## Results

### Participants

Among 12 participants who enrolled in this study, one participant withdrew from the study because of a schedule conflict. Two participants were excluded because their behavioral data were not recorded due to a technical failure. In addition, one participant did not perform the finger tapping task in the No-RAS condition as practiced. Therefore, a total of eight participants (four women and four men) were included in the analyses. Among them, five participants had their PET scans with RAS-condition first. The demographic characteristics and their PET scan parameters were presented in Table 1. Music experiences varied from none (*N* = 2), to 1 year (*N* = 1), 5 years (*N* = 5), 9 years (*N* = 1), and 10 years (*N* = 1). The PET parameters including the amount injected, specific activity, and mass inject were not significantly different between conditions.

**Table 1:** Demographics and PET scan parameters of eight right-handed young healthy participants.

Demographics		
Age	27.25 ± 4.65	
Sex (men: women)	4:4	
Years of music experience	4.38 ± 3.9	
Beck depression inventory	3.75 ± 5.09	
PET scan parameters		

		***p*-value**

Amount injected (mCi)		
No-RAS condition	9.27 ± 1.04	*p* = 0.601
RAS condition	9.80 ± 0.58	
Specific activity (mCi/μmol)		
No-RAS condition	1323.54 ± 425.62	*p* = 0.732
RAS condition	1315.65 ± 254.56	
Mas injected (μg)		
No-RAS condition	1.85 ± 0.45	*p* = 0.616
RAS condition	1.89 ± 0.31	

*Data are presented in mean and standard deviation.*

### Normality of Outcome Measures

The Shapiro–Wilk test did not show any significant results in normality of BP_ND_ values. Therefore, the statistical analyses were conducted using parametric tests.

### Finger Tapping Performance

**FIGURE 2:**
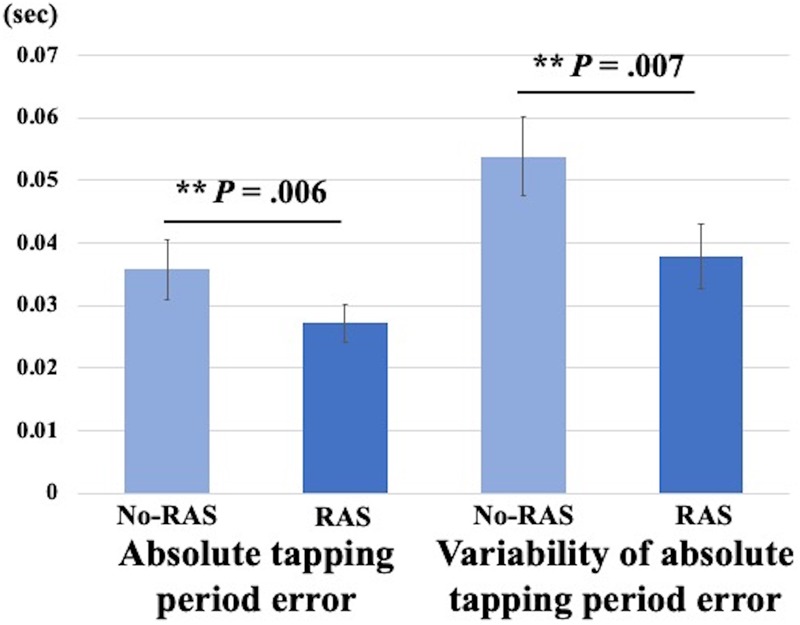
Mean differences in finger tapping performance between No-RAS and RAS conditions. In the RAS condition, both absolute tapping period error and its variability were significantly reduced compared to in the No-RAS condition. Error bars represent standard deviation.

Rhythmic auditory stimulation significantly improved the finger tapping task performance in young healthy individuals (Figure 2). Two-sided paired *t*-tests revealed that the absolute tapping period error was significantly reduced with RAS compared to without RAS (0.027 ± 0.009 vs. 0.036 ± 0.014, *t*(7) = 3.8, *p* = 0.007). Similarly, the variability of the absolute tapping period error was significantly reduced with RAS compared to without RAS (0.038 ± 0.015 vs. 0.052 ± 0.018, *t*(7) = 3.9, *p* = 0.006). The better finger tapping performance in the RAS-condition was consistently found across the participants regardless of the scan order. The number of outliers did not significantly differ between conditions, indicating that the participants engaged equally in both tasks and that the number of taps did not affect the BP_ND_ differences (Wessel et al., 1997). The outliers accounted for approximately 2% of the finger tapping performance on average in each condition.

### BP_ND_ Values

**FIGURE 3:**
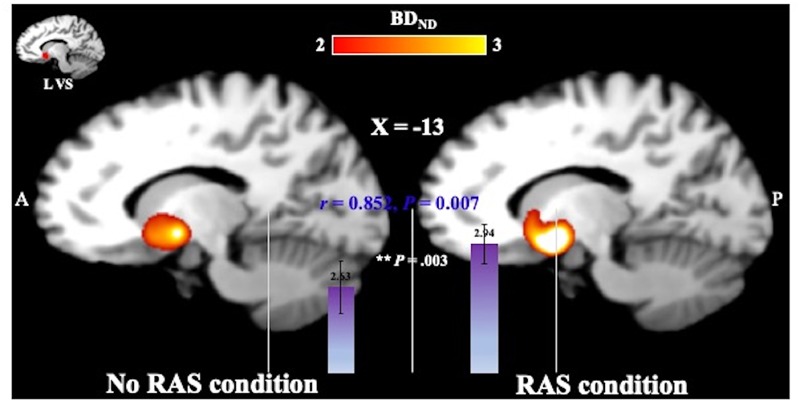
Mean BP_ND_ images in the left ventral striatum (L VS) in MNI space for each condition generated using SPM for visualization. The ROI mask was shown in the image in the upper left corner. In the RAS condition, BP_ND_ was significantly higher compared to in the No-RAS condition, suggesting that RAS was associated with less DA responses. Error bars represent standard error of the mean.

**FIGURE 4:**
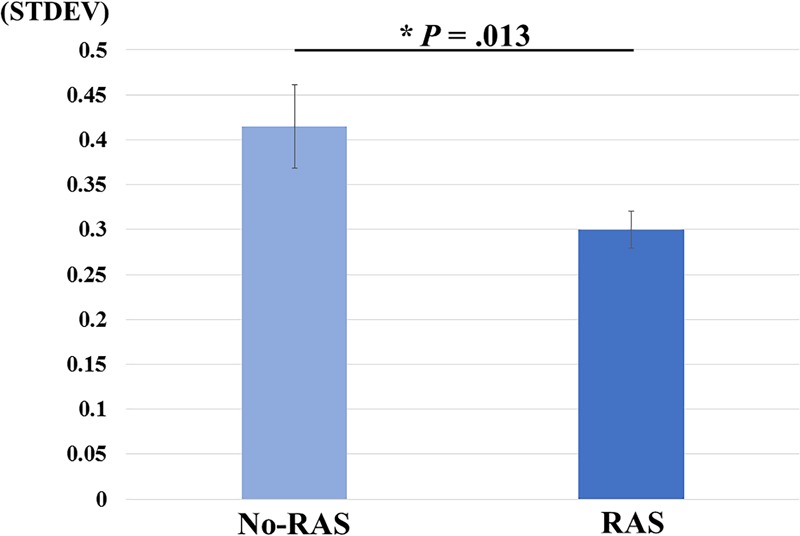
Mean differences in BP_ND_ variability changes between No-RAS and RAS conditions across the participants and regions of interest. In the RAS condition, the variability was significantly reduced compared to in the No-RAS condition. Error bars represent standard error of the mean. STDEV: standard deviation.

A two-sided paired *t*-test did not reveal a significant difference in the BG BP_ND_ value between the task conditions. However, the BP_ND_ value was significantly higher in the RAS condition compared to in the No-RAS condition in the left VS (2.942 ± 0.407 vs. 2.625 ± 0.541; *t*(7) = 4.515, *p* = 0.003), indicating less DA responses in the RAS condition compared to in the No-RAS condition in this region (Figure 3). The higher BP_ND_ values in the left VS in the RAS-condition was also consistently found across the participants regardless of the scan order. There were no significant differences in any other ROIs. Because RAS significantly reduced behavioral variability, we also explored whether the variability in BP_ND_ value in the entire BG (measured by the standard deviation across the participants and regions) also differed between conditions. Similar to the behavioral result, the RAS condition resulted in significantly less variability in BP_ND_ value in the entire BG compared to the No-RAS condition (0.3 vs. 0.42, *t*(7) = 3.289, *p* = 0.013) (Figure 4).

### Correlations Between BP_ND_ Values in the Left Ventral Striatum and Finger Tapping Performance

To further investigate the DA responses in the left VS, we first performed a Pearson correlation analysis to investigate the confounding effects of age and music experience on finger tapping performance and BP_ND_ values in the BG regions. Age showed significant negative correlations with the BP_ND_ values in the left putamen in the No-RAS condition (*r* = -0.77, *p* = 0.026) and in the right VS in the RAS-condition (*r* = -0.789, *p* = 0.02). Because there were no confounding effects on the BP_ND_ values in the left VS, we used a bivariate correlation to test (1) between BP_ND_ values in the left VS and finger tapping performance for each condition and (2) between the percentage change of the BP_ND_ values in the left VS and of finger tapping performance between conditions. No significant correlations were found.

## Discussion

This is the first study to investigate DA responses with [^11^C]-(+)-PHNO PET during auditory-motor entrainment using RAS in young healthy adults. Our major findings include that the presentation of RAS significantly improved finger tapping task performance and that the presentation of RAS led to significantly reduced DA responses in the left VS. In addition, increasing age was associated with reduced D2/3 R availability in the right VS in the RAS-condition and in the left putamen in the No-RAS condition.

As we hypothesized, the presentation of RAS improved finger tapping task performance in young healthy adults as indicated by reductions of absolute tapping period error and its variability measured using the standard deviations. This is consistent with previous literature (Rao et al., 1997; Jantzen et al., 2005) and corroborated the positive effects of RAS on motor behaviors (Ghai et al., 2018). Consistent with our hypothesis, BP_ND_ values would significantly different between conditions in BG. More specifically, the absence of RAS resulted in a significantly greater DA response in the left VS. The VS is part of the mesolimbic dopamine pathway and is implicated to play an important role in reward and motivational processing (Richard et al., 2013; Berridge and Kringelbach, 2015; Saga et al., 2017). The significant finding in the left laterization in the VS may be associated with its functional connectivity with the default mode network (DMN) (Zhang et al., 2017). DMN becomes activated in the thought process in which attention is internally directed such as episodic memory retrieval and planning (Spreng, 2012). Therefore, the absence of RAS may have reflected more motivational/attentional efforts directed toward the internal control of motor timing without auditory rhythmic cueing. Contrary to our hypothesis, finger tapping performance was not associated with dopaminergic function. This may be because the motor responses are more associated with cortical motor areas or cortico-subcortical connectivity.

In addition to these major findings, less D2/3 R availability in the left putamen was associated with increasing age in the No-RAS condition. The striatal D2/3 R availability is highly age-sensitive, as demonstrated in younger healthy adults (Yang et al., 2003). Furthermore, young healthy adults who display the D2R polymorphism associated with reduced D2 R availability (Jönsson et al., 1999) showed increased striatal activation during a perceptual timing task (Wiener et al., 2014). Therefore, less D2/3 R availability in the No-RAS condition observed in our data may be an age associated compensatory response for a more challenging/effortful task condition. These suggest that individual variance in D2/3 R availability due to age and the polymorphism needs to account for in the future studies.

Increasing age was also associated with less D2/3 R availability in the right VS in the RAS condition. Music modulates brain activity in the NAc/VS (Menon and Levitin, 2005; Salimpoor et al., 2011). The right laterization may be associated with rightward processing of music (Menon and Levitin, 2005; Zatorre et al., 2007), musical pleasure (Menon and Levitin, 2005; Salimpoor et al., 2011), reward (Martin-Soelch et al., 2011), and/or emotion (Molochnikov and Cohen, 2014). It is unknown why increased age was associated with less D2/3 R availability in the right VS in the RAS condition as there is no literature to support this finding. DA changes in the mesocorticolimbic system associated with reward and motivation may partially contribute to some of the age-related motor performance (Seidler et al., 2010). Further investigations are needed including a larger number of participants as well as including self-reports on emotion and valence concerned with RAS.

The current study has shed light on the roles of DA in auditory-motor entrainment using RAS in a small sample size of young healthy adults. The initial findings warrant future studies to confirm and further elucidate the current findings with a larger sample size of younger and older healthy adults as well as persons with dopamine-based movement disorders such as PD. If RAS can modulate DA responses in PD, it is of particular interest because the dopaminergic role in the enhancement of motor control in PD with RAS is unknown. The knowledge of baseline D2/3 R availability will allow to determine whether or not the tasks induce significant DA release and will also help to interpret the significant reduction in the variability of DA responses in BG with RAS. It was interesting to find that both neural and behavioral variability measures decreased in the RAS condition although a direct correlational analysis could not be done due to the different metric calculations. A future study can address whether the presence of RAS would modulate individual DA functions to behave similarly. In addition, the knowledge of genetic variations in the D2/3 R subtypes will be of use. As a significant difference in DA response was observed in the VS that is associated with musical pleasure, reward, and motivation, the administration of associated self-reports regarding RAS may be able to further clarify the interpretations of the current findings.

## Author Contributions

YK, AS, S-sC, and MT conceived and designed the study. YK, MV, and VS acquired the data. YK analyzed the data. YK and MT interpreted the data. YK drafted the manuscript. AS, MV, VS, S-sC, SH, and MT critically reviewed the manuscript.

## Conflict of Interest Statement

The authors declare that the research was conducted in the absence of any commercial or financial relationships that could be construed as a potential conflict of interest.
